# Dosimetric evaluation and clinical application of collimated apertures with proton beam line scanning in stereotactic radiotherapy

**DOI:** 10.1002/acm2.70128

**Published:** 2025-05-29

**Authors:** Chen‐Yu Chou, Hsiao‐Chieh Huang, Shen‐Hao Lee, Shih‐Ming Hsu

**Affiliations:** ^1^ Department of Radiation Oncology Linkou Chang Gung Memorial Hospital Taoyuan City Taiwan (R.O.C); ^2^ Department of Biomedical Imaging and Radiological Sciences National Yang Ming Chiao Tung University Taipei City Taiwan (R.O.C)

**Keywords:** apertures, pencil beam line scanning, stereotactic radiotherapy

## Abstract

**Purpose:**

Stereotactic radiotherapy (SRT) is a highly effective treatment with precision for small, localized lesions. Proton therapy, characterized by the Bragg peak, offers superior dose conformity compared to photon‐based approaches. However, challenges remain in minimizing lateral penumbra and optimizing dose delivery, particularly for small targets. This study presents the first clinical application of collimated apertures integrated with the proton line scanning technique for proton stereotactic radiotherapy (PSRT). The aim was to evaluate the dosimetric advantages and clinical feasibility of this innovative approach.

**Methods:**

Over a 1‐year period, 30 patients with small lesions, including choroid melanoma and arteriovenous malformations, were treated using proton line scanning. Two planning strategies were evaluated: uncollimated proton line scanning (UPLS) and collimated proton line scanning (CPLS), incorporating patient‐specific apertures. Dosimetric comparisons were conducted using the Homogeneity Index (HI), Paddick Conformity Index (CI_Paddick_), Gradient Index (GI), and R50%. Treatment accuracy was validated using absolute dose measurements and Gamma Passing Rate (GPR) analysis under the criteria of 3%/3 and 2%/2 mm.

**Results:**

Plans incorporating customized collimated apertures showed significant improvements in dose conformity, with higher CI_Paddick_ values (*p* < 0.001), and exhibited steeper dose fall‐off, as reflected in lower GI and R50% values (*p* < 0.001). A trend toward more homogeneous dose distributions was also observed (*p* < 0.001). GPR analysis confirmed high treatment accuracy, with an average value of 99.00 ± 1.83% (3%/3 mm) and 91.06 ± 4.91% (2%/2 mm).

**Conclusions:**

Integrating customized collimated apertures with proton beam line scanning is clinically feasible, improving precision, dose conformity, and healthy tissue sparing in PSRT. These findings support adopting this novel approach to advance precision proton therapy for small lesions.

## 1 INTRODUCTION

Precision radiotherapy has transformed cancer care, with stereotactic radiotherapy (SRT) and stereotactic radiosurgery (SRS) emerging as necessary tools for noninvasive treatment of small, well‐defined lesions. These techniques enable the delivery of high radiation doses with sub‐millimeter precision, thereby minimizing radiation exposure to healthy tissues and improving patient outcomes. Despite advancements in treatment delivery systems, achieving optimal dose conformity and steep dose gradients for small targets remains an ongoing challenge, particularly in anatomically complex regions.

Photon‐based techniques, while widely implemented, are inherently limited by the physical characteristics of photon beams, resulting in unintended irradiation of surrounding healthy tissues. In contrast, proton therapy leverages the Bragg peak phenomenon, enabling highly localized radiation with a sharp distal fall‐off beyond the target. This unique energy deposition profile minimizes unnecessary exposure to adjacent critical structures and reduces integral dose to healthy tissues, thereby lowering the risk of radiation‐induced toxicity. Additionally, proton beams exhibit a higher linear energy transfer (LET) within the Bragg peak region, potentially enhancing biological effectiveness in tumor control. Compared to photon‐based approaches, proton therapy provides superior dose conformity and allows for precise modulation of dose gradients, which is particularly advantageous for small lesions located near critical structures. Among proton delivery methods, pencil beam scanning (PBS) offers enhanced intensity modulation, enabling tailored dose distribution with improved normal tissue sparing compared to conventional passive scattering techniques. These dosimetric advantages make proton therapy an optimal choice for stereotactic radiotherapy applications, where maximal dose precision is required.

Despite the advantages of PBS, its application in small‐target treatments is restricted by the inherent limitation of lateral penumbra, which compromises dose conformity and spatial precision. This lateral penumbra arises primarily from multiple Coulomb scattering as protons pass through tissues, leading to increased beam broadening, particularly at shallow depths. To address this issue, several studies explored the integration of collimated apertures with PBS to reduce lateral spread and enhance dose delivery.^1^, ^2^, ^3^, ^4^, ^5^, ^6^, ^7^, ^8^, ^9^, ^10^, ^11^, ^12^, ^13^ Most studies employed spot scanning or passive scattering techniques, leaving the potential of proton line scanning (PLS) largely unexplored. Unlike spot scanning, which delivers proton beams to discrete points within the target, PLS utilizes a continuous beam motion along a predefined trajectory across the target. The method offers theoretical advantages in efficiency and dose homogeneity.^14^, ^15^, ^16^, ^17^, ^18^

This study represents the first clinical application of custom‐made collimated apertures integrated with line scanning for proton stereotactic radiotherapy (PSRT). Although the previous study^19^ primarily focused on commissioning, feasibility testing, and the verification of medical performance, the present work extends the investigation to the clinical implementation of real patient treatment. By systematically evaluating treatment precision, dose conformity, and spatial dose gradients in patient cases, this study provides critical insights into the practical efficacy of this approach.

## 2 MATERIAL AND METHODS

### 2.1 Patient population

This study reports the clinical results of a year‐long investigation following the commissioning of a custom‐made collimated aperture system integrated with line scanning for PSRT. Thirty patients with small, well‐defined targets were treated at the hospital institute. The target lesions included choroid melanoma, arteriovenous malformations (AVM), schwannomas, and meningiomas located in critical anatomical regions such as the ocular region, midbrain, temporal lobe, and cerebellopontine angle. These targets posed significant challenges due to their size and proximity to critical organs.

Target volumes ranged from 0.51 to 35.81 cm^3^ (mean: 5.81 cm^3^, standard deviation: 8.14 cm^3^), with prescribed doses varying between 12 and 50 Gy (RBE) delivered in 1–5 fractions. The cohort reflects the diversity and complexity of small‐target stereotactic radiotherapy cases, underscoring the need for an innovative approach capable of achieving high precision and steep dose gradients. Detailed information on the thirty cases analyzed in this study is presented in Table 1.

**TABLE 1:** Comprehensive summary of 30 cases in the current research.

Patient	Diagnosis	Location	Volume (cc)	Dose (Gy (RBE)]	Fraction	Optimization^a^	Fields^b^
1	Acoustic neuroma	Left CPA^c^	18.34	18	3	SFO	1G115 2G180 3G100C310
2	AVM^d^	Right midbrain	0.57	21	3	SFO	1G270 2G90 3G300C90
3	AVM	Right temporal	21.53	22.5	3	MFO	1G310 2G250 3G280C40
4	AVM	Right basal ganglion	1.24	13	1	SFO	1G260 2G260C30 3G260C60 4G260C90
5	AVM	Midbrain‐pontine vascular lesion	0.55	18	3	SFO	1G220 2G140 3G220C90
6	AVM	Midbrain	4.26	18	3	MFO	1G120 2G240 3G0
7	AVM	Midbrain	4.26	18	3	SFO	1G90 2G270 3G300C90
8	Cavernoma	Left superior frontal cavernous lesion	9.19	21	3	SFO	1G0 2G90 3G310C90
9	Cavernoma	Right pons	18.04	15	3	SFO	1G270 2G90 3G300C90
10	Choroid melanoma	Right eye	2.05	50	5	SFO	1G300 2G300C20 3G255
11	Choroid melanoma	Right eye	1.04	50	5	MFO	1G10 2G300 3G320C90
12	Choroid melanoma	Right eye	0.87	50	5	SFO	1G300 2G300C60
13	Choroid melanoma	Right eye	0.93	50	5	SFO	1G240 2G300C40 3G300C90
14	Choroid melanoma	Right eye	0.51	25	5	SFO	1G280C60 2G280C30 3G265
15	Choroid melanoma	Left eye	2.37	50	5	MFO	1G95 2G60 3G60C300
16	Choroid melanoma	Right eye	2.71	50	5	SFO	1G250 2G300C40 3G270C90
17	Choroid melanoma	Left eye	0.75	18	3	MFO	1G55 2G60C340 3G100
18	Choroid melanoma	Left eye	3.17	50	5	SFO	1G100 2G65 3G65C320
19	Choroid melanoma	Right eye	2.11	50	5	MFO	1G255 2G255C40 3G270C65
20	Choroid melanoma	Left eye	2.06	50	5	SFO	1G80 2G110 3G80C300
21	Choroid melanoma	Left eye	2.7	50	5	SFO	1G105 2G40C320 3G270C90
22	Choroid melanoma	Right eye	1.36	50	5	SFO	1G270C10 2G250C30 3G250C60
23	Choroid melanoma	Left eye	3.75	50	5	SFO	1G90 2G130C330 3G120C300
24	Choroid melanoma	Right eye	4.08	50	5	MFO	1G245 2G300C90 3G330C40
25	Choroid melanoma	Right eye	2.12	50	5	SFO	1G260 2G270C40 3G270C90
26	Meningioma	Left superior parietal convexity	2.87	21	3	SFO	1G330C90 2G270C90 3G270C60
27	Meningioma	Left parasellar (skull base)	16.6	15	3	SFO	1G60C300 2G70 3G100
28	Schwannoma	Left CPA	4.93	12	1	SFO	1G155 2G45 3G60C330
29	Schwannoma	Left CPA	35.81	50	5	SFO	1G100 2G180 3G100C330
30	Schwannoma	Right CPA	3.53	21	3	SFO	1G200 2G310 3G330C20

^a^ MFO stands for multiple‐field optimization. SFO stands for single‐field optimization.

^b^ G stands for gantry angle. C stands for couch angle.

^c^ CPA stands for cerebellopontine angle.

^d^ AVM stands for arteriovenous malformation.

### 2.2 Treatment planning and delivery

Proton treatments were delivered using a P235 cyclotron (Sumitomo Heavy Industries) with line scanning mode. Treatment plans were developed with the Eclipse Treatment Planning System (v13.7, Varian Medical Systems, Palo Alto, CA), integrating the PCS_PBSRS calculation model for optimized dose calculation and delivery. The algorithm allows dose rates up to 1045 MU/s and supports both single‐field optimization (SFO) and multi‐field optimization (MFO) approaches. SFO was predominantly used for its simplicity and robustness, while MFO was reserved for more geometrically complex targets.

This study presents a comparative analysis of two treatment approaches: UPLS and CPLS. The UPLS group underwent treatment using the line scanning technique alone, without a collimated aperture, whereas the CPLS group incorporated a custom‐designed aperture system integration with line scanning. This comparison aimed to evaluate the impact of collimated apertures on dose conformity, spatial precision, and the reduction of low‐dose exposure to surrounding normal tissues. Furthermore, this study investigates whether integrating collimated apertures into treatment planning leads to an improvement in overall plan quality, thereby enhancing the therapeutic efficacy of proton therapy for small‐target treatments.

To enhance treatment robustness against setup and range uncertainties, robust optimization was employed, incorporating a 2 mm setup uncertainty and a 3.5% range uncertainty. These parameters were selected based on institutional data, which reflect the observed patient setup variability at the institute. Twelve perturbation scenarios were generated by applying translational shifts along all principal axes, which were then incorporated into the worst‐case optimization framework. The primary objective of the treatment planning process was to achieve complete target volume coverage within the 100% prescribed isodose line, ensuring consistent and homogeneous dose distribution across the target.

To enhance dose precision and mitigate lateral penumbra, a collimated aperture system was integrated into the conventional proton line scanning treatment workflow. The system consists of three key components: (1) a primary collimator with a 9‐cm physical diameter upstream of the beam to refine beam geometry, (2) a 4‐cm thick snout degrader for range modulation, specifically used for superficial targets, and (3) a 6‐cm thick custom brass aperture placed downstream, individually designed for each patient to shape the beam and reduce dose spread.

To further optimize dose conformity and improve therapeutic efficacy, the aperture margin was determined through Beam's Eye View (BEV) of each treatment field. A 5 mm isotropic expansion beyond the target boundary was applied to ensure adequate target coverage while minimizing unnecessary radiation exposure to surrounding healthy tissues.^19^

**FIGURE 1:**
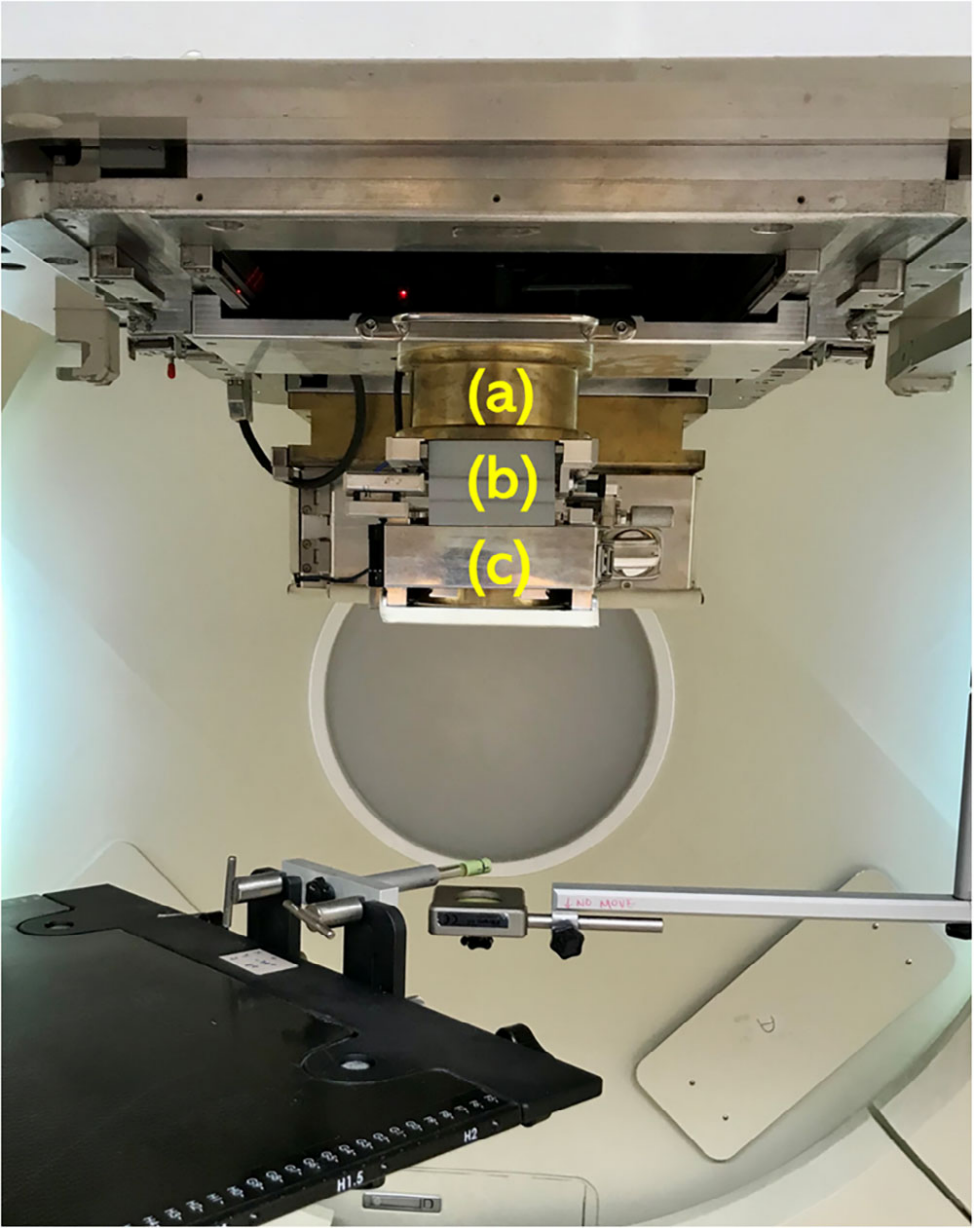
The components of the collimated aperture device include (a) a primary collimator positioned upstream and (b) an intermediate snout degrader, with (c) a patient‐specific aperture located downstream.

**FIGURE 2:**
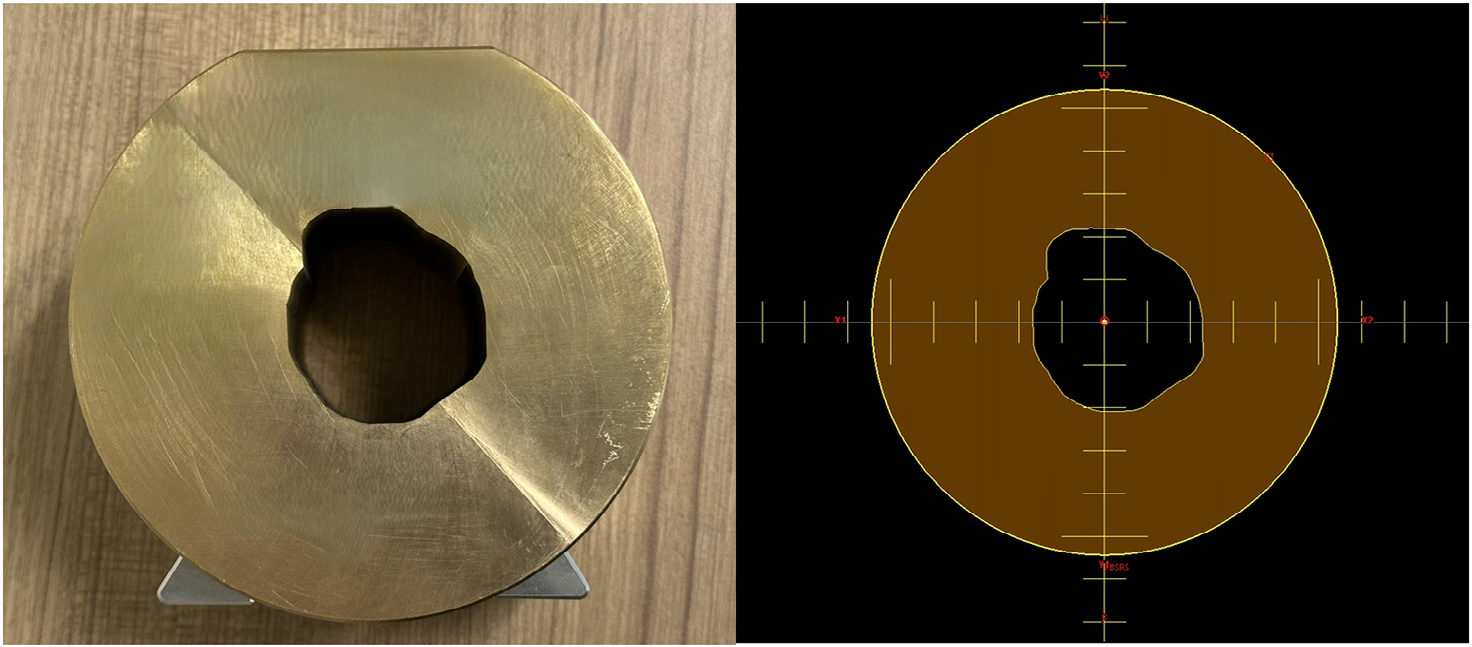
An example of a photograph and field design of an aperture based on the target geometry of a treatment field.

The system supports field sizes at the isocenter ranging from 1–6 cm, enabling clinical implementation across different cases. Figure 1 demonstrates the geometric configuration of the aperture system. Figure 2 presents an example of an aperture photograph and field design tailored to the target geometry of a specific treatment field.

### 2.3 Dosimetric evaluation and measurement

The homogeneity index (HI) was used to evaluate the uniformity of dose distribution across various treatment plans, ensuring similarity in plan quality between those utilizing the collimated aperture and those without. Although HI is often considered of minor importance in SRS and SRT, its evaluation is crucial for the overall quality and robustness of the treatment plan. By identifying potential hot spots and cold spots, it minimizes the risk of side effects arising from treatment uncertainties. Additionally, HI allows for comparing different treatment plans and techniques, facilitating the selection of the most optimal approach. The calculation of the Homogeneity Index involves dividing the minimum dose in 5% of the Target volume (D_5_) by the minimum dose in 95% of the target volume (D_95_).^20^

(1) $$HI=\frac{D_{5}}{D_{95}}$$

The evaluation of conformity to the prescribed dose distribution utilized the Paddick Conformity Index (CI_Paddick_) as proposed in ICRU Report 91.^21^, ^22^, ^23^ This index is defined as

(2) $$CI_{Paddick}=\frac{{\left(TV_{PIV}\right)}^{2}}{TV\times PIV}$$

where TV_PIV_ stands for the target volume covered by the prescription isodose volume, TV is the target volume, and PIV represents the prescription isodose volume.

The conformity and homogeneity indices are important for analyzing treatment plans in radiation therapy. The primary objective in CPLS planning was to achieve treatment outcomes equivalent or superior to those achieved with UPLS plans. This involved delivering the prescribed therapeutic dose with high conformity and homogeneity to the target volume while minimizing exposure to surrounding normal tissue and critical organs.

To systematically evaluate clinical toxicity risks, several key factors were analyzed, including dose fall‐off, the radiation dose received by critical organs, and the potential for radiation‐induced necrosis. A crucial aspect of minimizing radiation exposure outside the target area is the steepness of the dose gradients. This steepness was quantitatively assessed using the Gradient Index (GI). In this context, the GI is a significant metric, defined as the ratio between the volume encompassed by the 50% prescription isodose line (V_50_) and the volume within the prescription isodose line (PIV). This ratio provides a clear measure of how sharply the radiation dose diminishes beyond the targeted treatment zone, thereby helping to optimize therapeutic efficacy while reducing collateral damage to surrounding healthy tissues.^21^

(3) $$GI=\frac{V_{50}}{PIV}$$

The final parameter utilized for plan evaluation is R50%, employed to assess the low‐dose regions.^24^

(4) $$R50\%\;=\frac{V_{50}}{TV}$$

Treatment planning quality assurance encompasses absolute dose measurement and dose distribution evaluation. To measure the absolute doses of small fields, a PTW (Physikalisch‐Technische Werkstaetten, Freiburg, Germany) 31014 Pinpoint chamber with an active volume of 0.015 cm^3^ was utilized.

For absolute dose verification, the treatment plan was applied to the water phantom imaging dataset to compute the dose distribution for each treatment field. A clinically relevant measurement location was identified, and the absolute dose predicted by the treatment planning system (TPS) was systematically documented for comparative dosimetric evaluation. The proton beam was subsequently delivered to the phantom, and the ion chamber‐measured dose, corrected for relevant calibration factors, was quantitatively compared against the TPS‐predicted dose to ensure dosimetric accuracy and verification.

For two‐dimensional (2D) dose distribution evaluation, a similar procedure was followed, except that Gafchromic EBT3 radiochromic film was placed at the measurement depth within the phantom. Following irradiation, the films underwent analysis using the Gamma Passing Rate (GPR) in FilmQA Pro software (Ashland Inc., Wayne, NJ, accessible at http://www.filmqapro.com) to assess the agreement between the measured dose distribution and TPS calculations.

## 3 RESULTS

### 3.1 Plan evaluation

Thirty cases were included in this study, as detailed in Table 1. The therapeutic dose varied from 12 Gy (RBE) to 50 Gy (RBE), depending on the tumor type. The beam arrangement mostly contained three fields, with only one case necessitating four fields to achieve the clinical objective.

**FIGURE 3:**
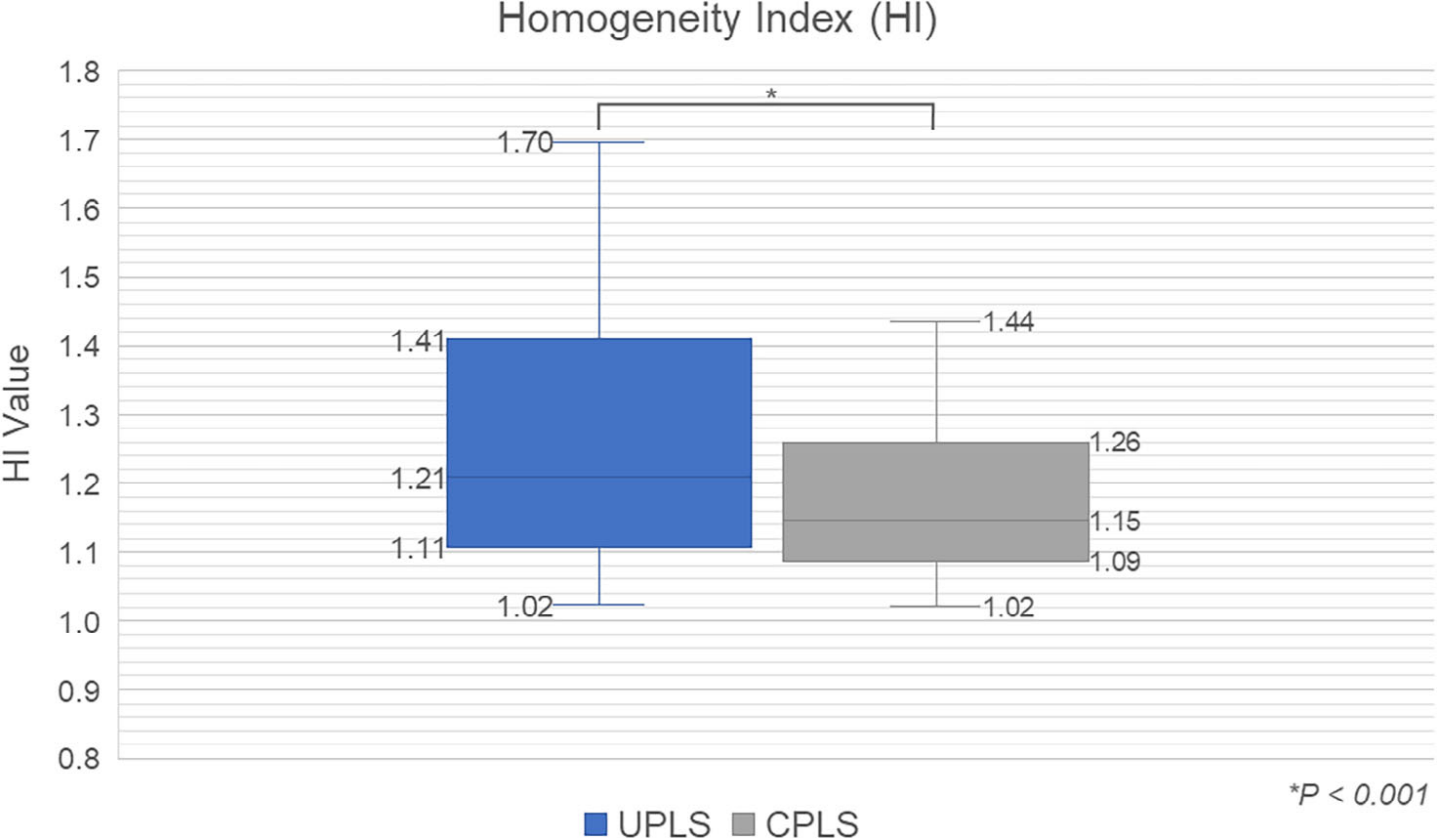
Comparison of Homogeneity Index (HI) values between UPLS and CPLS. CPLS, collimated proton line scanning; HI, homogeneity index; UPLS, uncollimated proton line scanning.

Figure 3 shows the boxplot of HI values for the study. The differences in HI between UPLS and CPLS were minimal, reflecting the consistent clinical objectives and optimization strategies applied in both techniques. However, the figure indicates a trend toward improved dose homogeneity in the CPLS group. Paired t‐test analysis demonstrated a statistically significant enhancement in homogeneity for CPLS (*p* < 0.001). The enhanced dose homogeneity observed in the CPLS group, while ensuring adequate target coverage, can be ascribed to the implementation of a customized collimated aperture system. This system effectively mitigates unintended dose deposition to adjacent critical structures, thereby optimizing beam fluence uniformity. Consequently, the reduced dependence on highly modulated beam intensities enhances the efficiency and robustness of treatment delivery, reinforcing the clinical feasibility of aperture‐based PSRT.

**FIGURE 4:**
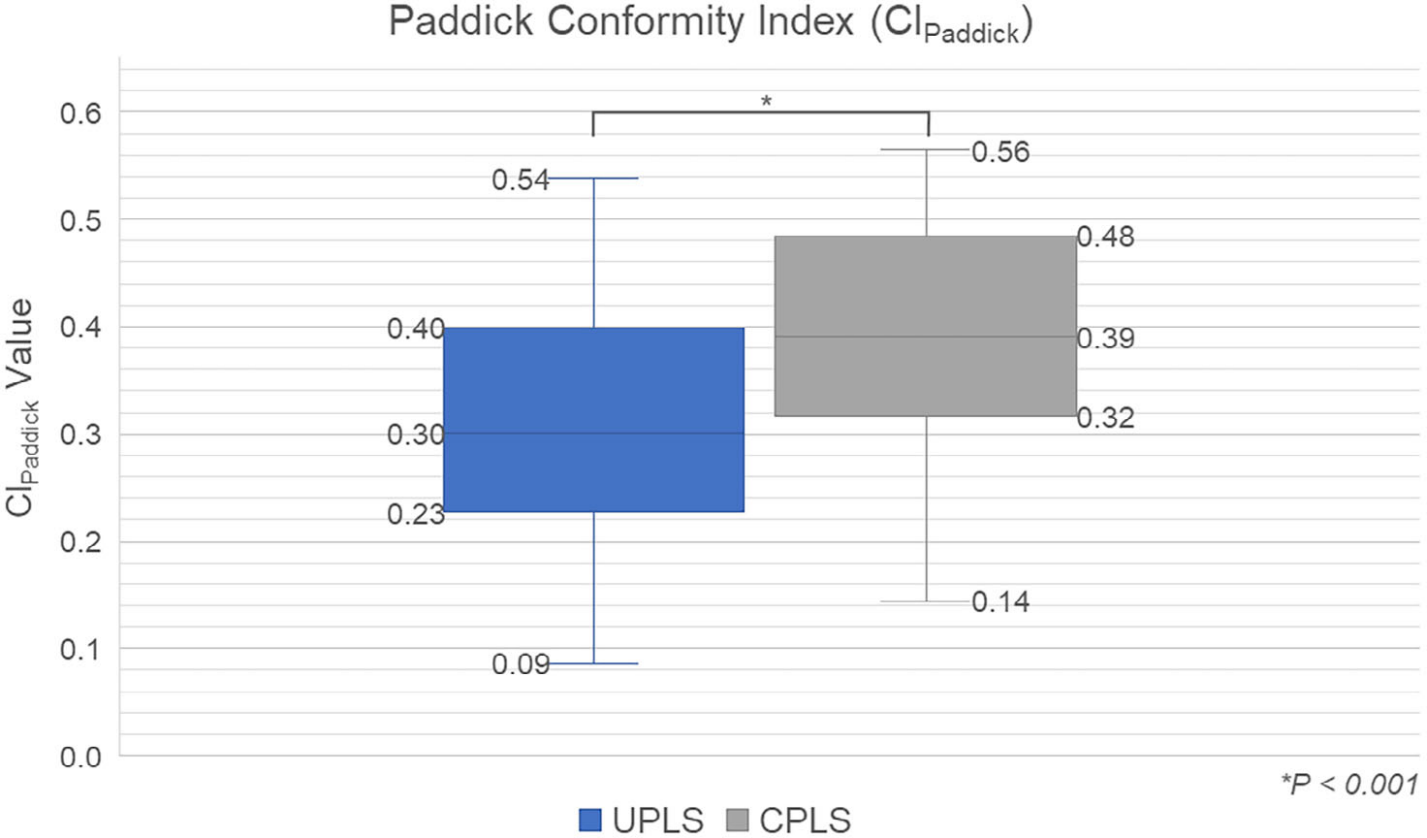
Comparison of Paddick Conformity (CI_Paddick_) values between UPLS and CPLS. CPLS, collimated proton line scanning; UPLS, uncollimated proton line scanning.

Figure 4 depicts the comparison of the CI_Paddick_ for the entire study cohort, as detailed in Equation 2. A CI_Paddick_ value approaching one indicates superior target dose conformity. As shown in the figure, CPLS plans achieved statistically significant superiority over UPLS plans regarding the conformity index (*p* < 0.001).

**FIGURE 5:**
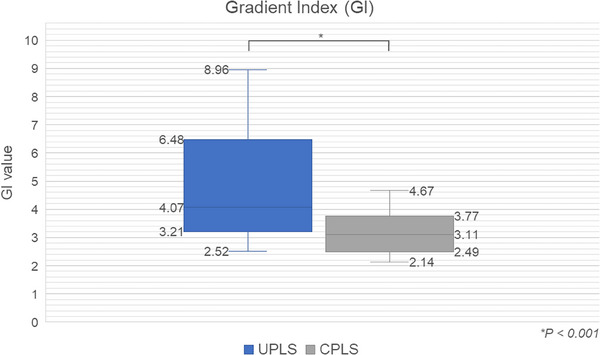
Comparison of Gradient Index (GI) values between UPLS and CPLS. CPLS, collimated proton line scanning; UPLS, uncollimated proton line scanning.

**FIGURE 6:**
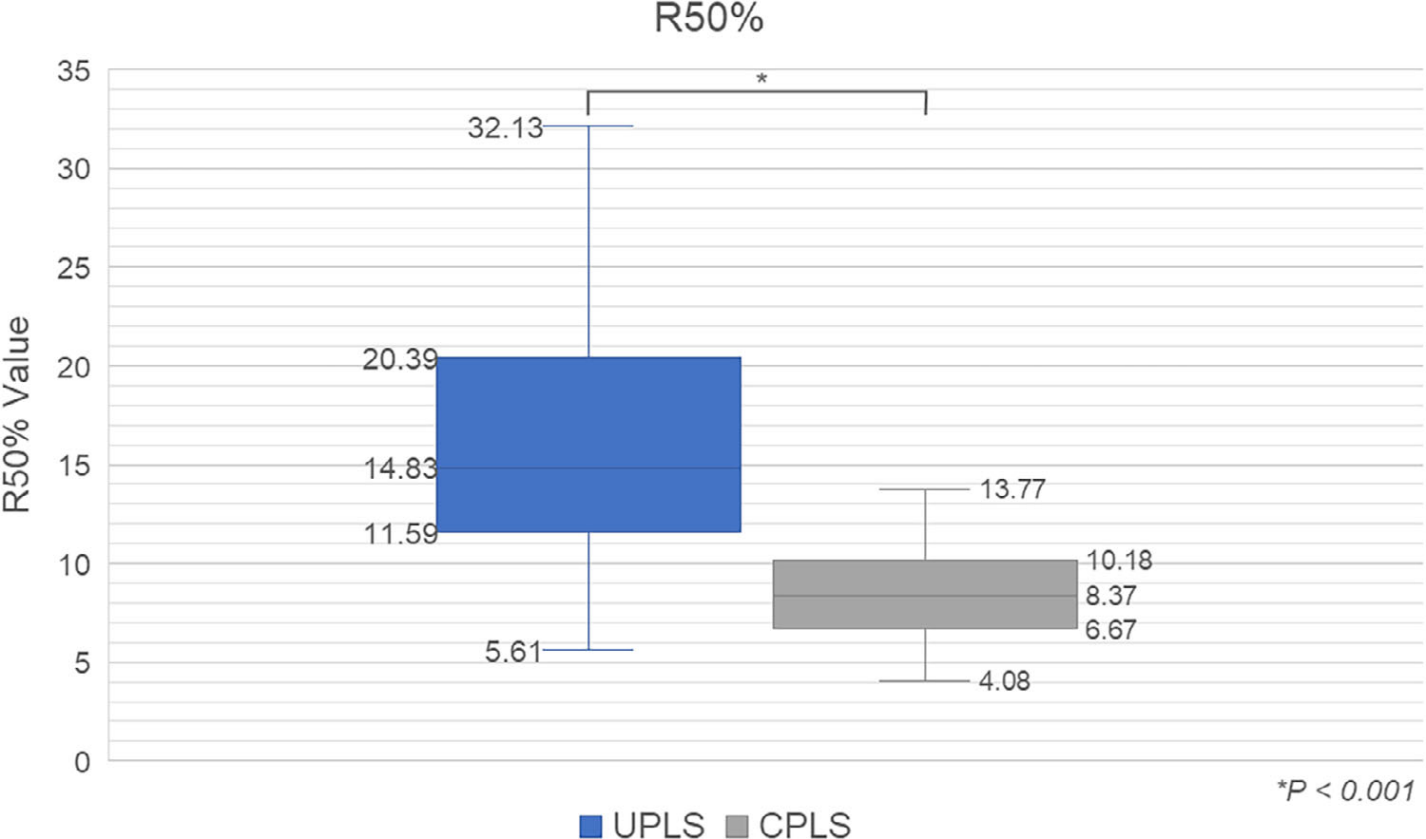
Comparison of R50% values between UPLS and CPLS. CPLS, collimated proton line scanning; UPLS, uncollimated proton line scanning.

The GI and R50% values are depicted in Figures 5 and 6. The data consistently demonstrate the superior performance of CPLS plans, as evidenced by significantly lower GI and R50% values (*p* < 0.001). These findings, evident in both GI and R50% values, support that the aperture configuration employed in CPLS plans facilitates a sharper dose fall‐off. This translates to a reduction in the low‐dose region near the target boundary. Consequently, this configuration minimizes collateral damage to surrounding healthy tissues and critical organs.

The evaluation of conformity and homogeneity indices demonstrated that CPLS planning achieved superior dose distribution characteristics compared to UPLS plans. The incorporation of collimated apertures in line scanning resulted in enhanced dose conformity, ensuring precise target coverage while maintaining dose homogeneity within the target volume. The use of collimated apertures effectively reduced the lateral penumbra, thereby minimizing radiation exposure to surrounding normal tissues. The reduction in low‐dose spread contributed to improved normal tissue sparing, particularly in proximity to critical structures.

### 3.2 Dosimetric verification

This study utilized two approaches for dosimetric verification, employing both absolute dose measurements and the GPR analysis. Absolute dose measurements were conducted using a PTW 31014 pinpoint ion chamber to assess concordance between treatment planning system (TPS) calculations and delivered dose.

**FIGURE 7:**
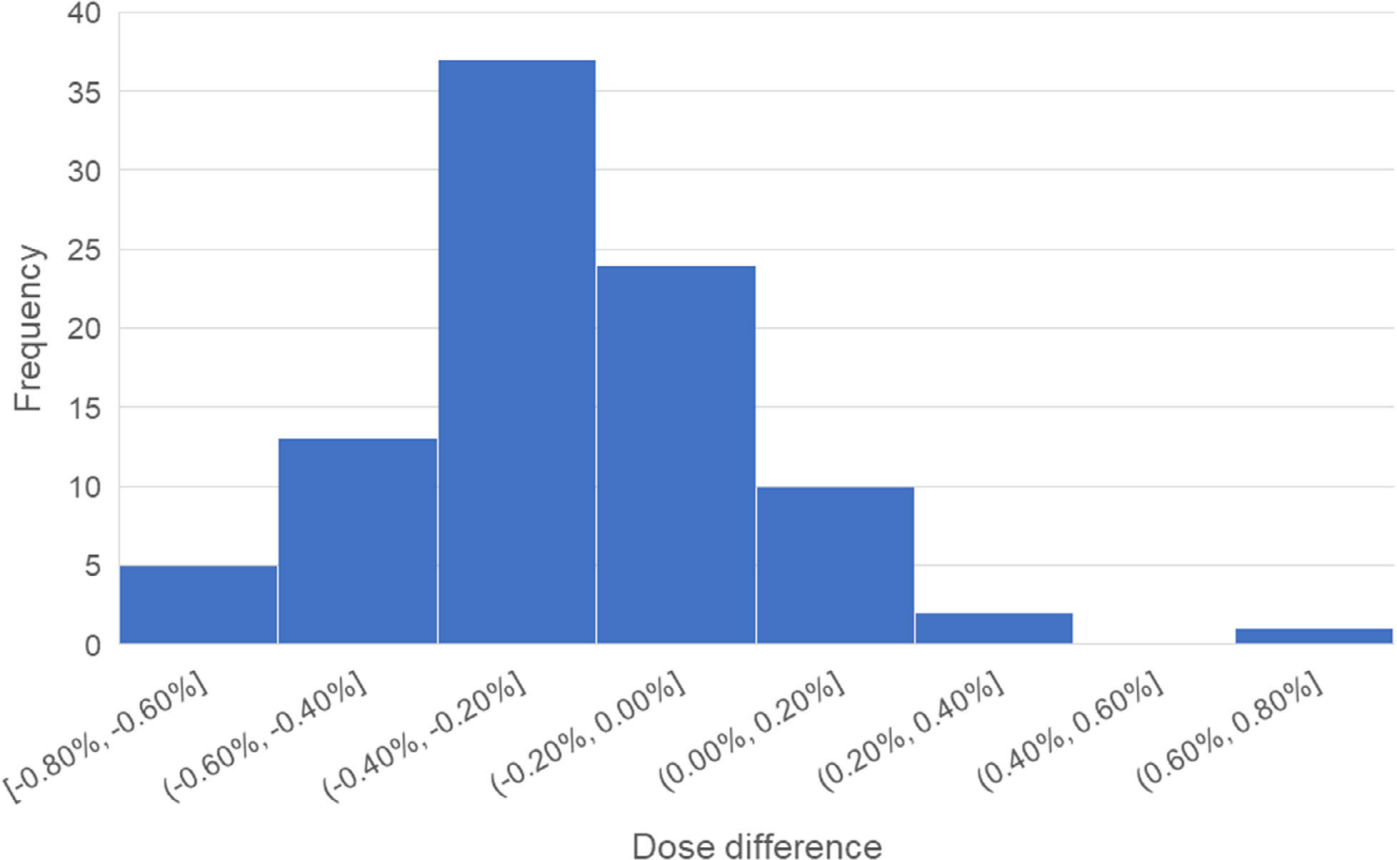
Distribution of dose difference between plan and measurements of all treatment fields.

The results demonstrated a high degree of agreement between calculated and measured doses. The dose discrepancy remained within ± 1.00% for all 30 patients, encompassing a total of 90 treatment fields assessed at the isocenter point. The mean dose difference was ‐0.23 ± 0.23%, signifying a negligible systematic bias. Figure 7 shows the histogram of dose discrepancies.

**FIGURE 8:**
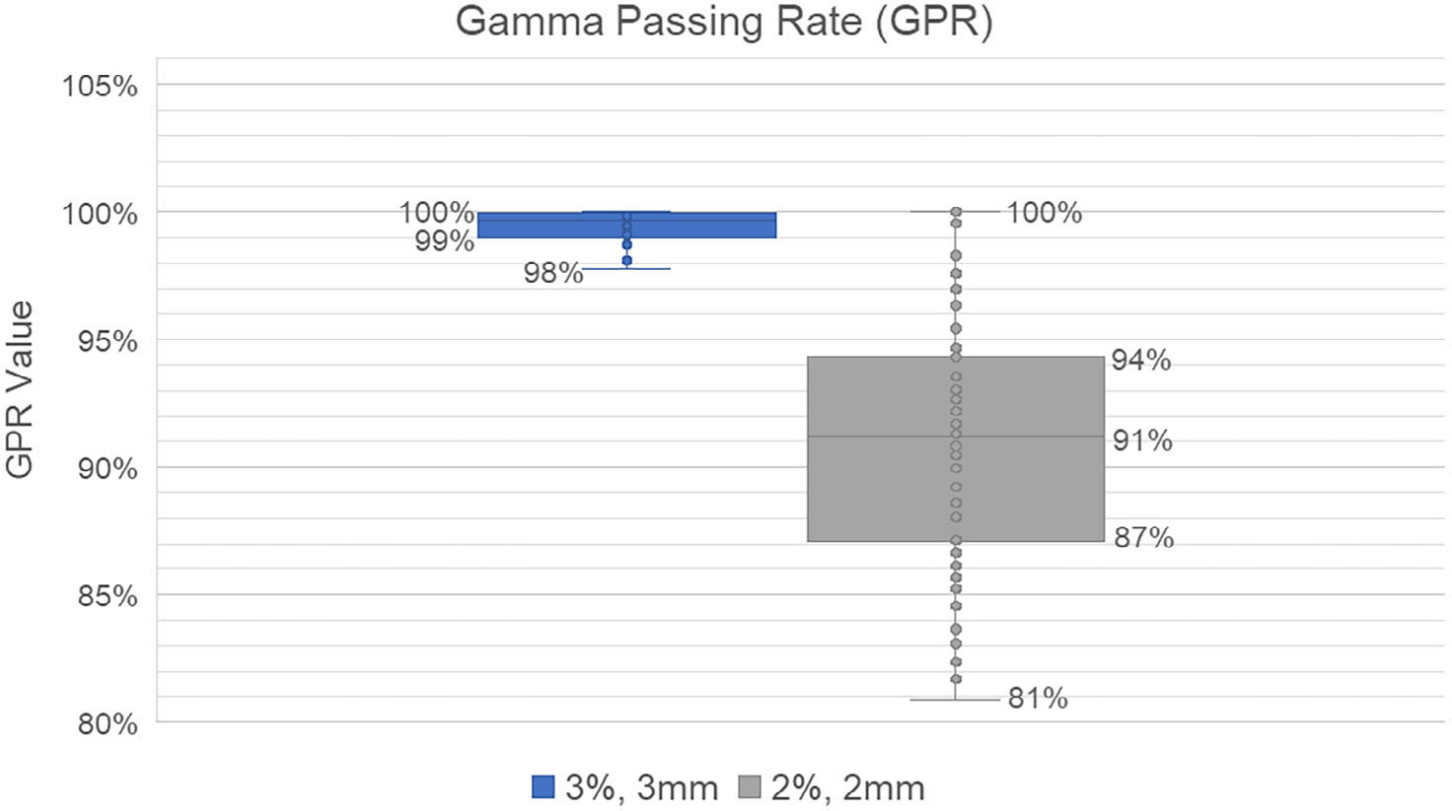
Gamma passing rate (GPR) value comparison for 3%, 3 mm, and 2%, 2 mm.

The GPR served as a quantitative measure of agreement between the measured and planned dose distributions. Utilizing the commonly employed criteria of 3%/3 mm, the average GPR achieved a high value of 99.00 ± 1.83%. Even under the stricter criteria of 2%/2 mm, the average GPR remained exceptionally high at 91.06 ± 4.91%. These consistently high planar GPR values signify a strong correlation between the measured and planned dose distributions. Figure 8 visually depicts the GPR values across the entire treatment fields.

In this study, the GPR analysis was conducted with a 10% dose threshold to account for the computational limitations of the pencil beam algorithm within the treatment planning system.^25^, ^26^, ^27^, ^28^, ^29^, ^30^ This threshold was applied to mitigate the inaccuracies of pencil beam dose calculations in low‐dose regions, particularly those outside the primary treatment field when a collimated aperture was employed. By restricting the analysis to regions receiving at least 10% of the prescribed dose, the evaluation focused on clinically relevant high‐dose areas. This approach provides a more representative validation of the TPS's performance in confined irradiation fields for stereotactic proton therapy of small‐volume lesions.

**FIGURE 9:**
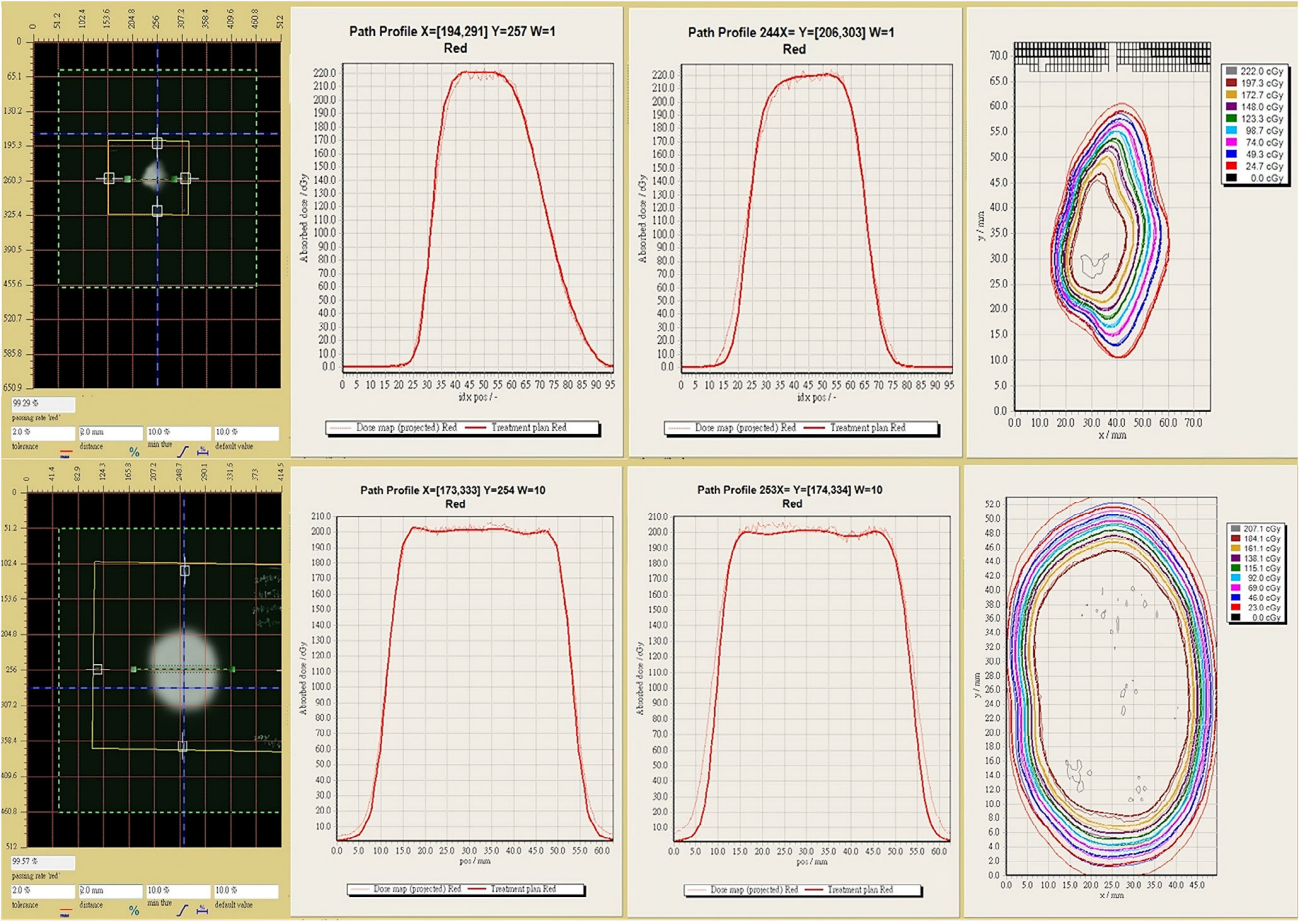
Examples of profile and Gamma Passing Rate (GPR) analysis for different target volumes.

Although most cases demonstrated GPR values exceeding 90% under the 2%/2 mm criteria, a limited number of cases demonstrated slightly reduced passing rates, typically from 80% to 90%. These deviations were primarily observed in treatment plans targeting volumes adjacent to critical structures such as the eye, optic nerves, and other critical organs. In such cases, strict dose constraints were applied to minimize the risk of radiation‐induced toxicity. Consequently, modifications to aperture design were often required to balance target coverage with critical organ sparing. These modifications occasionally introduced steep dose gradients and complex geometries, challenging both dose modeling and delivery verification. Nonetheless, all treatment plans satisfied clinical acceptability criteria, and the localized GPR reductions did not compromise the overall therapeutic objectives or treatment reliability. Figure 9 illustrates representative cases of dose profile comparisons and corresponding GPR analyses across different target volumes.

## 4 DISCUSSION

This study systematically evaluated the feasibility and dosimetric advantages of integrating a static customized‐brass aperture into a proton therapy system, with a specific focus on its implementation in PSRT. The adoption of this approach was strategically driven by the objective of minimizing disruptions to established clinical workflows while avoiding the need for extensive beam nozzle modifications. To facilitate seamless integration, an accessory holder was developed to accommodate both the static brass aperture and the snout degrader, providing a practical solution that maintains the operational integrity of existing treatment protocols.

The implementation of a static aperture in PSRT demonstrated several potential advantages over the conventional line scanning technique. First, there was a visible trend towards more homogeneous dose distributions within the CPLS group, indicating the aperture's capacity to optimize dose distribution and reduce the necessity for intensive beam modulation, particularly in scenarios involving critical structures close to the target volume. Furthermore, CPLS plans yielded statistically significant improvements in CI_Paddick_ values, signifying superior spatial precision in dose sculpting around the target volume. Additionally, reductions in the GI and R50% values were observed, indicative of a more rapid dose fall‐off beyond the target periphery. This sharper dose gradient contributed to improved critical organ sparing and enhanced normal tissue preservation, underscoring the dosimetric benefits and clinical feasibility of the aperture‐based PSRT approach.

Dosimetric validation, performed through absolute dose measurements and GPR analysis, further supported the precision, reproducibility, and clinical reliability of the proposed approach. The close agreement between treatment planning system calculations and measured dose values, combined with GPR results meeting established criteria for conventional proton plans, confirms the reliability of integrating collimated apertures within a proton beam line scanning framework. This high level of dosimetric consistency is critical for maintaining treatment accuracy, minimizing the risk of target underdosing, and mitigating unnecessary radiation exposure to adjacent normal tissues, thereby optimizing both therapeutic efficacy and patient safety.

Despite its dosimetric advantages, the clinical implementation of a static collimating aperture introduces several technical and operational challenges that warrant careful consideration. As a high atomic number (high‐Z) material, brass contributes to secondary neutron generation and radiation‐induced activation, necessitating thorough radiation protection strategies within clinical environments. The resulting secondary neutron contamination may require additional shielding measures and extended post‐irradiation cooldown periods to ensure radiation safety for clinical personnel re‐entering the treatment room. From an operational standpoint, the integration of patient‐specific apertures introduces logistical complexities, including prolonged manufacturing lead times, dedicated storage infrastructure, and additional pre‐treatment QA protocols. These QA measures include isocenter verification, structural integrity assessments of aperture devices, image‐guided localization accuracy evaluations, and comprehensive safety interlock system verifications before treatment. Addressing these considerations is critical to ensuring the seamless clinical translation of aperture‐based proton therapy while maintaining strict safety and workflow efficiency standards.

Although the incorporation of collimated apertures in proton therapy presents dosimetric advantages, their clinical implementation necessitates a careful balance between therapeutic benefit and logistical feasibility. The findings of this study underscore the potential of aperture‐based proton therapy to improve dose conformity and treatment precision for small, well‐localized lesions. However, successful clinical translation requires careful workflow integration, including considerations for manufacturing logistics, quality assurance protocols, and radiation safety measures. Addressing these operational challenges will be essential to fully leverage the benefits of collimated apertures in proton therapy while maintaining treatment efficiency and compliance with standard clinical practice.

## 5 CONCLUSION

This study demonstrates the clinical feasibility and dosimetric advantages of integrating collimated apertures with proton pencil beam line scanning for stereotactic radiotherapy of small, well‐localized targets. The implementation of a static brass aperture enhanced dose conformity and homogeneity, as evidenced by statistically significant improvements in the HI and CI_Paddick_. Furthermore, the steeper dose gradient achieved, as indicated by lower GI and R50% values, effectively minimized radiation exposure to adjacent critical organs while maintaining target dose coverage.

Dosimetric validation, including absolute dose measurements and GPR analysis, confirmed the accuracy, reproducibility, and reliability of this approach, supporting its clinical applicability. The collimated aperture reduced the need for excessive beam modulation, thereby improving delivery efficiency. Additionally, the use of an aperture improved lateral penumbra control, which is beneficial for treating small lesions requiring high‐dose precision.

These findings demonstrate aperture‐based PSRT stereotactic radiotherapy as a practical and effective strategy for improving treatment plan quality in proton therapy for small‐volume lesions, with the potential to enhance both therapeutic efficacy and normal tissue sparing.

Despite its dosimetric benefits, the introduction of a high‐Z collimating material introduces operational and radiological safety considerations, including secondary neutron production, radiation‐induced activation, and logistical challenges related to aperture manufacture and quality assurance. Future studies can focus on further refining the clinical workflow, optimizing aperture design for variable field sizes, and evaluating long‐term clinical outcomes in larger patient cohorts.

## AUTHOR CONTRIBUTION

**Chen‐Yu Chou**: Conceptualization, methodology; investigation; data collection; Formal Analysis; Writing—Original Draft. **Hsiao‐Chieh Huang**: Project administration; coordination; **Shen‐Hao Lee**: Project administration; coordination. **Shih‐Ming Hsu**: Supervision; Writing—Review & Editing

## CONFLICT OF INTEREST STATEMENT

The authors declare no conflicts of interest.
